# Radiation Exposure in Robotic-Assisted Versus Conventional and Navigation-Assisted Spine Surgery: A Systematic Review, Meta-Analysis, and Meta-Regression of 3205 Patients

**DOI:** 10.3390/jcm15062144

**Published:** 2026-03-11

**Authors:** Víctor Rodríguez-Domínguez, Catalina Vivancos Sánchez, Mario Taravilla-Loma, María L. Gandía-González, Alberto Isla Guerrero

**Affiliations:** 1 Department of Neurosurgery, Fundación Jiménez Díaz University Hospital, 28040 Madrid, Spain; 2 Department of Neurosurgery, Rey Juan Carlos University Hospital, 28933 Madrid, Spain; 3 Neurosurgery Department, La Paz University Hospital, 28046 Madrid, Spainmtaravillaloma@gmail.com (M.T.-L.); neurocirugiagandia@gmail.com (M.L.G.-G.)

**Keywords:** radiation, robotic, freehand, navigation, spine surgery, meta-analysis

## Abstract

**Objectives**: This systematic review and meta-analysis compared freehand and navigation-assisted spine surgery with robot-assisted techniques, focusing on radiation dose, fluoroscopy time, and factors influencing these outcomes. **Methods**: Following the PRISMA and PROSPERO protocols, we searched major databases for comparative studies on radiation exposure or fluoroscopy duration. Non-comparative, cadaveric, and animal studies were excluded. Bias was assessed with RoB 2 and MINORS. The data were pooled using random-effects models, with subgroup, meta-regression, sensitivity, and publication-bias analyses. **Results**: Twenty-eight studies (3205 patients) were included. Compared with freehand surgery, robotic assistance did not significantly reduce radiation dose (SMD −0.81; *p* = 0.07) or fluoroscopy time (SMD −0.56; *p* = 0.06), with substantial heterogeneity. Subgroup analyses revealed lower exposure with specific robotic systems (e.g., Tianji^®^), in degenerative and trauma indications, and at cervical, lumbar, and thoracolumbar levels. No differences were observed between robotic-assisted and navigation-assisted techniques. A meta-regression showed increasing an fluoroscopy time and radiation dose in more recent freehand studies, while trends were stable in robotic cohorts. No publication bias was detected. **Conclusions**: Robotic-assisted surgery was not associated with statistically significant reductions in overall radiation dose or fluoroscopy time compared with freehand techniques. Effects may vary by robotic platform, indication, and anatomical level; however, substantial heterogeneity limits certainty. Further randomized controlled trails with standardized reporting are warranted.

## 1. Introduction

Surgical integration, particularly in robotics, is transforming multiple surgical domains and has gained relevance in spinal procedures [1]. Robotic systems offer precise control, minimally invasive approaches, and the potential for improved outcomes [2]. Nevertheless, their use in spine surgery remains debated, especially regarding radiation exposure [3,4]. Evidence suggests that robotic assistance may decrease radiation exposure for patients and operating room staff due to enhanced precision and potentially shorter operative times [5]. However, this assumption is not universally supported [6,7]. Several studies have questioned whether robotics truly reduces radiation, emphasizing the need for more rigorous evaluation [8]. While some publications report meaningful reductions in radiation levels [9], others describe no difference or even increased exposure [10].

Recent systematic reviews and meta-analyses provide inconsistent findings. Luengo-Matos [11] included only four studies addressing radiation reduction, and Li et al. [12] found no significant differences in fluoroscopy time between robotic and freehand techniques, based on merely two studies. Similarly, Guan et al. [13] reported no differences in overall radiation exposure, though subgroup analysis with six studies suggested lower doses in robotic-assisted TLIF compared with MIS-TLIF. Al-Naseem et al. [14] also detected no significant differences, relying on only three studies.

Given these limitations, a more comprehensive systematic review and meta-analysis is required to clarify whether robotic assistance meaningfully affects radiation exposure and how potential confounders influence reported outcomes.

## 2. Materials and Methods

### 2.1. Protocol Registration

This systematic review and meta-analysis adhered to the PRISMA (Preferred Reporting Items for Systematic Reviews and Meta-Analyses) statement [15] (Supplementary Table S1) and the recommendations of the Cochrane Handbook for Systematic Reviews and Meta-Analyses [16]. The protocol for this review was published and registered in PROSPERO with ID CRD42024608425.

### 2.2. Data Sources and Search Strategy

We searched the Cochrane Central Register of Controlled Trials (CENTRAL), PubMed (MEDLINE), Web of Science (WoS), and SCOPUS databases from inception until October 2024, with a reminder in all databases turned on to identify peer-reviewed eligible studies using search terms with Boolean operators. ClinicalTrials.gov was also searched for trial registration (Table A1).

### 2.3. Eligibility Criteria

The PICO criteria guided study selection: population (patients undergoing spinal surgery, including minimally invasive pedicle screw placement and spinal fusions), intervention (robotic-assisted technique), comparison (freehand or navigation-assisted), and outcomes (radiation dose and fluoroscopy time). We excluded reviews, editorials, book chapters, cadaveric/animal studies, and non-comparative studies without radiation data.

### 2.4. Study Selection

The Rayyan tool was used for screening. All retrieved records were imported into Rayyan (Version 1.7, [Computer software]. (2026). Rayyan Systems, Inc., Doha, Qatar) for de-duplication and screening. After removing the duplicates, two authors independently assessed all records using the blinding feature and then screened the full texts of potentially relevant studies. Rayyan labels/tags were used to record exclusion reasons and key notes; filters were used to track decisions by stage and reviewer and to export a reproducible decision log. Disagreements were resolved by consensus. Forward and backward citation tracking was also performed for the included studies.

### 2.5. Data Extraction

The study data were exported to Excel and organized by study characteristics, patient demographics, and outcomes (radiation dose and fluoroscopy time). The variables included the study design, indication, robot type, sample size, screws used, age, sex, and BMI. The radiation dose reflected cumulative ionizing exposure; fluoroscopy time captured X-ray duration. Radiation exposure and fluoroscopy time were extracted exactly as reported in each eligible study. Because radiation exposure was reported using different dosimetry measures and units (e.g., dose–area product, air kerma, effective dose, or occupational dose), and fluoroscopy time was reported with different time bases (e.g., per procedure, per screw, or during instrumentation) and units (seconds/minutes), we synthesized outcomes using standardized mean differences (SMD). To improve transparency and reproducibility, we summarized each study’s radiation dose measure/unit and fluoroscopy time basis in Table A3; when a study did not report a field, it was coded as “NA”.

### 2.6. Risk of Bias

The RCTs were assessed with the Cochrane RoB 2 tool [17] across six bias domains. Observational studies were evaluated using MINORS (0–24 scale: 0–6 is deficient, 7–10 is low, 11–15 is fair, 16–24 is high-quality) [18]. Two authors independently rated all studies, resolving disagreements through discussion with the senior author.

### 2.7. Statistical Analysis

All analyses were performed in R 4.3 using “meta,” “metafor,” and “dmetar.” the Standardized mean differences with 95% CIs were pooled using a DerSimonian–Laird random-effects model, with heterogeneity assessed via Chi-square and I^2^ following Cochrane criteria [16]. The comparisons included RA vs. FH and RA vs. NA. The subgroup and random-effects meta-regression analyses evaluated moderators. Meta-regression was conducted to formally explore potential sources of heterogeneity (e.g., publication year and other study-level moderators where available). Given the limited number of studies contributing to some moderators and the presence of residual heterogeneity, these analyses were considered exploratory. The sensitivity (leave-one-out) and Egger’s tests assessed robustness and publication bias [19].

## 3. Results

### 3.1. Search Results and Study Selection

**Figure 1:**
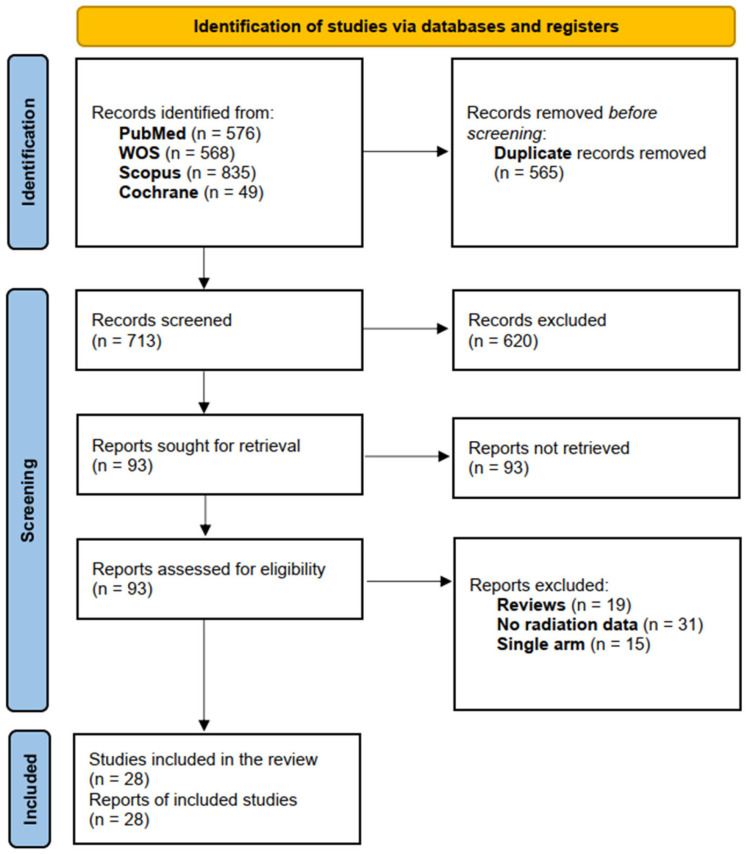
Study flow diagram.

By searching databases, 1278 records were retrieved. After duplicate removal, 713 references were left for primary screening by title and abstract. After screening by title and abstract, ninety-four articles were available to be assessed for our eligibility criteria. Finally, we included 28 studies enrolling 3205 patients in this systematic review and meta-analysis. The PRISMA flow chart of the selection process is shown in Figure 1.

### 3.2. Characteristics of the Included Studies

Most of the included studies were single-center studies, except for three that were multi-center [20,21,22]. Fourteen studies were conducted in China [20,23,24,25,26,27,28,29,30,31,32,33,34,35], five in the USA [21,22,36,37,38], six in Germany [10,39,40,41,42,43], one in Korea [44], one in Switzerland [45], and another in France [46]. Table 1 and Table 2 summarize the included studies and the baseline characteristics, respectively.

### 3.3. Risk of Bias

**Figure A1:**
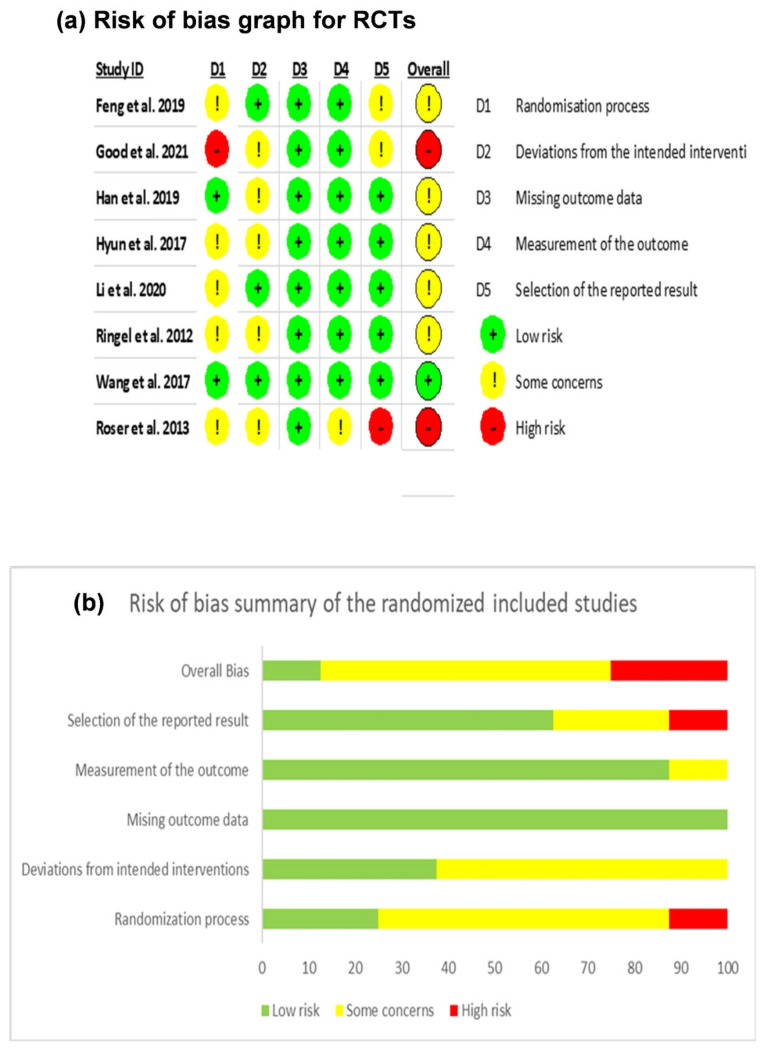
Risk of bias graph and risk of bias summary of RCTs.

Methodological quality was evaluated using RoB 2 for randomized trials (*n* = 8) and MINORS for non-randomized studies (*n* = 20). Most RCTs showed some concerns, with one low-risk study (Wang et al., 2017) [26] and two high-risk studies (Roser et al., 2013; Good et al., 2021) [10,22]. The MINORS scores (17–20/24) indicated high quality despite prospective-data limitations (Table A2, Figure A1).

### 3.4. Outcomes

#### 3.4.1. Radiation Dose

Robotic-assisted versus Freehand

**Figure 2:**
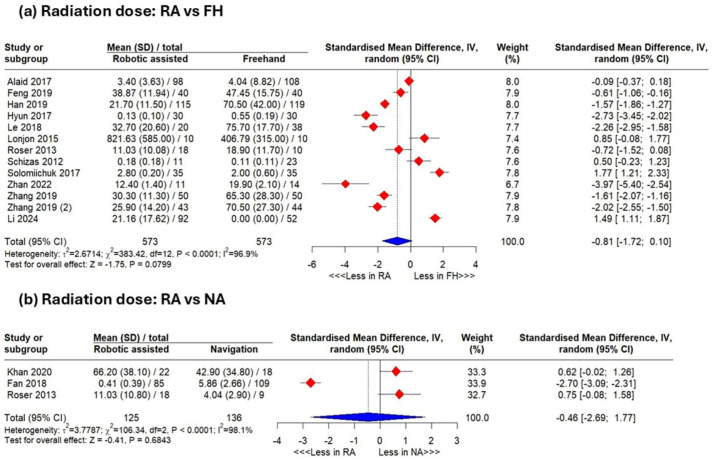
Results of meta-analysis. Analysis of radiation dose between the robotic-assisted group and the freehand group (**a**) and between the robotic-assisted and navigation-assisted group (**b**).

**Figure A2:**
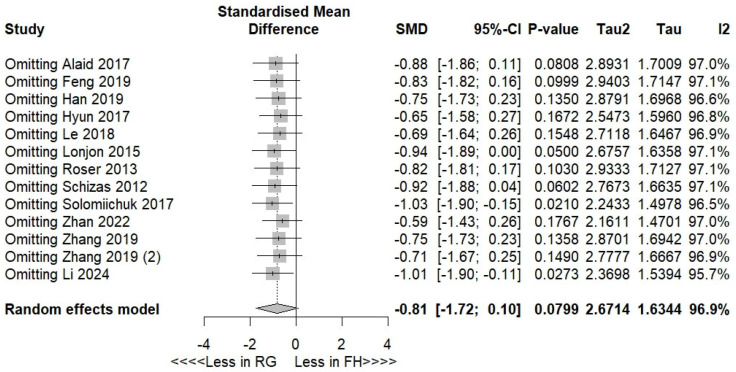
Sensitivity analysis by leave-one-out method for radiation dose.

With a high heterogeneity (I^2^ = 96.9%), the pooled results showed no significant radiation-dose difference between the robotic-assisted and freehand groups (SMD −0.81, 95% CI −1.83–0.21, *p* = 0.07) (Figure 2a). Sensitivity analyses excluding Solomiichuk et al. [43] or Li et al. [25] yielded significant reductions favoring robotics (*p* = 0.02) (Figure A2).

#### 3.4.2. Subgroup Analyses

**Figure 3:**
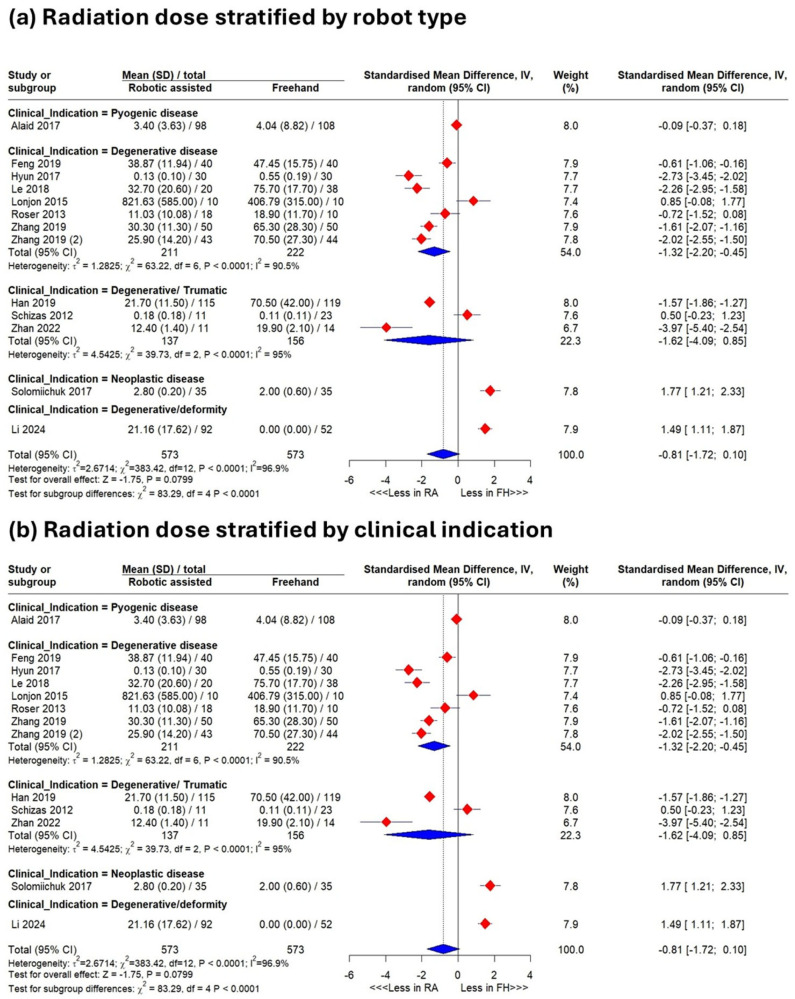
Results of meta-analysis. Analysis of radiation dose subgrouped according to type of robot (**a**) and clinical indication (**b**).

Subgroup analyses showed that Tianji^®^ (Tinavi) significantly reduced radiation dose (SMD −1.44, 95% CI −2.64–−0.25; *p* = 0.023) (Figure 3a). Grouped by indication, robotics reduced the dose in degenerative disease (SMD −1.32, 95% CI −2.20–−0.45), whereas neoplastic and degenerative/deformity clinical indications favored freehand techniques (1.77 [1.21–2.33]; 1.49 [1.11–1.87]; *p* < 0.0001) (Figure 3b).

**Figure 4:**
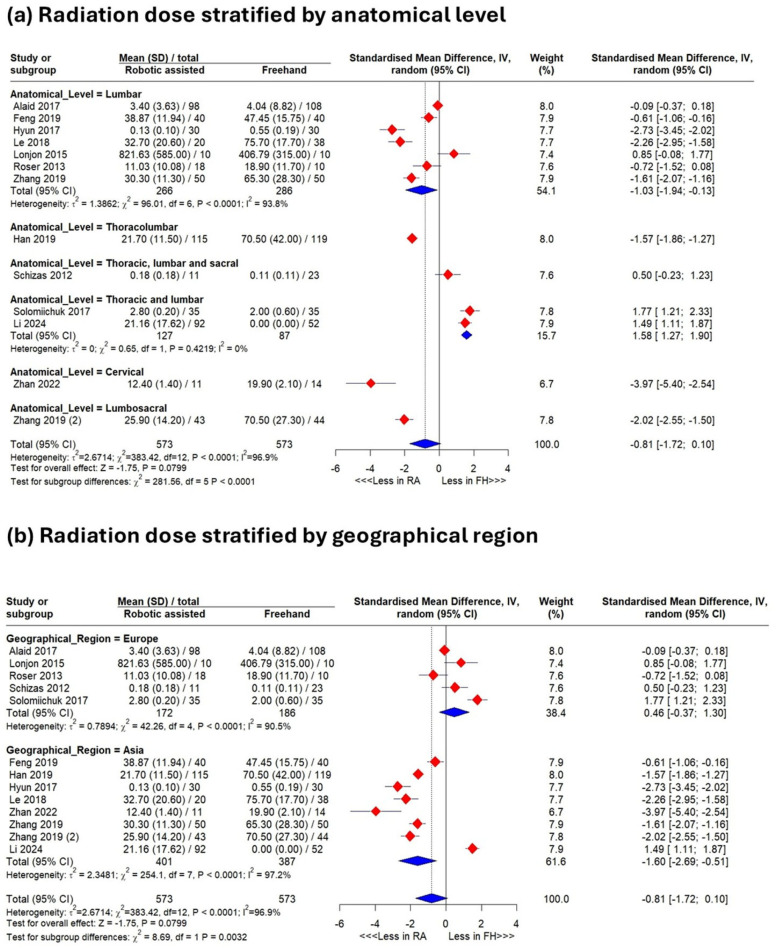
Results of meta-analysis. Analysis of radiation dose subgrouped according to anatomical level (**a**) and according to region (**b**).

Grouped by anatomical level, several regions favored robotics, but lumbar/thoracic-level procedures favored the freehand technique (SMD 1.58 [1.27–1.90]; *p* < 0.0001) (Figure 4a). Asian studies favored robotics (SMD −1.60 [−2.93–−0.28]; *p* = 0.003) (Figure 4b).

#### 3.4.3. Robotic-Assisted Versus Navigation-Assisted

Including heterogeneous studies (I^2^ = 98.1), the pooled analysis showed no significant difference between the robotic-assisted and navigation-assisted groups (SMD: −0.46 with 95% CI [−2.96, 1.77], *p* = 0.72) (Figure 2b).

#### 3.4.4. Fluoroscopy Time

Robotic-assisted versus Freehand

**Figure 5:**
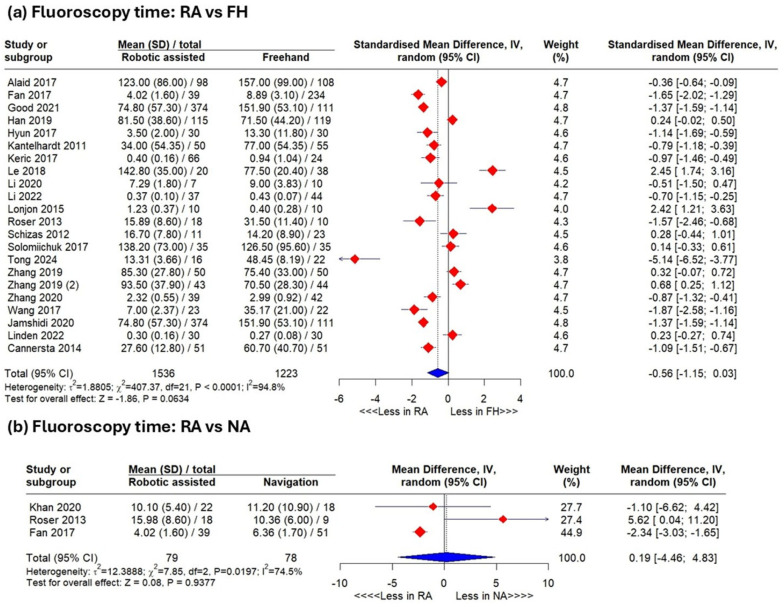
Results of meta-analysis. Analysis of fluoroscopy time between the robotic-assisted group and the freehand group (**a**) and between the robotic-assisted and navigation-assisted group (**b**).

**Figure A3:**
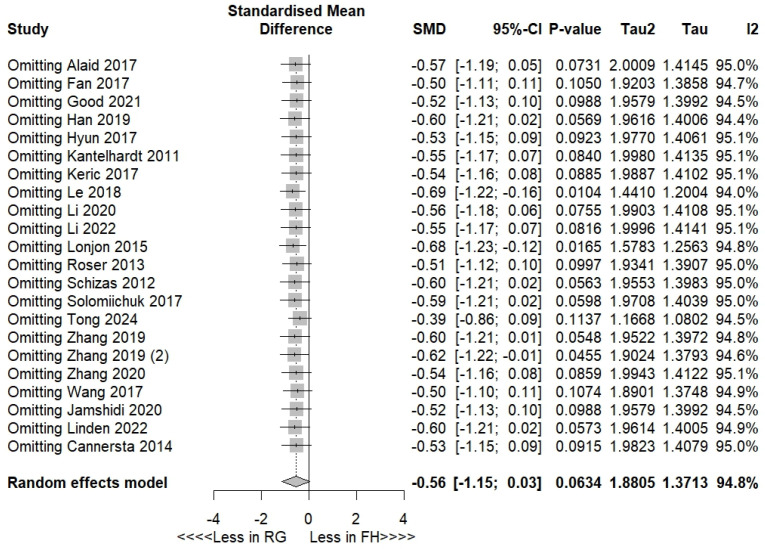
Sensitivity analysis by leave-one-out method for fluoroscopy time.

Including heterogeneous studies (I^2^ = 94.9%), the pooled analysis showed a trend toward lower fluoroscopy time in the robotic-assisted group versus freehand group (SMD −0.56, 95% CI −1.15–0.03; *p* = 0.06) (Figure 5a). Leave-one-out sensitivity analyses excluding Le et al. [29], Lonjon et al. [46], or Zhang et al. [34] demonstrated significantly reduced fluoroscopy time favoring robotics (Figure A3).

#### 3.4.5. Subgroup Analyses

**Figure 6:**
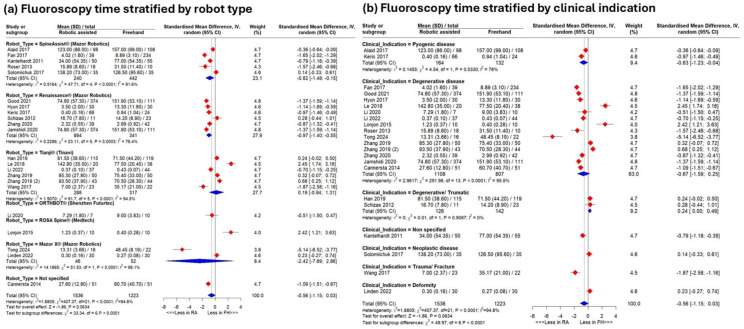
Results of meta-analysis. Analysis of fluoroscopy time subgrouped according to type of robot (**a**) and clinical indication (**b**).

**Figure A4:**
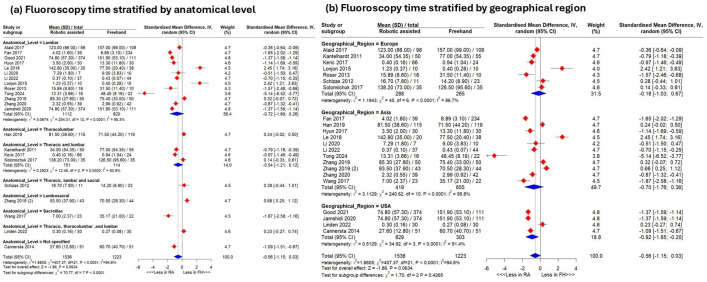
Fluoroscopy time stratified by anatomical level (**a**) and geographical region (**b**).

Subgroup analysis by robot type showed significantly lower fluoroscopy time for the Renaissance^®^ (SMD −0.97, 95% CI −1.40–−0.55) and Spine Assist^®^ (SMD −0.82, 95% CI −1.48–−0.15), whereas studies using the ROSA Spine^®^ favored freehand techniques (SMD 2.42, 95% CI 1.21–3.63; *p* < 0.0001) (Figure 6a). By clinical indication, pyogenic disease and trauma/fracture favored robotics (SMD −0.63 [−1.23–−0.04]; −1.87 [−2.58–−1.16]; *p* < 0.0001) (Figure 6b). Grouping by anatomical level showed a lower fluoroscopy time for procedures at a sacroiliac level (SMD −1.87 [−2.58–−1.16]), but a greater time for lumbosacral-level procedures (SMD 0.68 [0.25–1.12]; *p* < 0.0001) (Figure A4a). Regionally, USA studies favored robotics (SMD −0.92 [−1.65–−0.20]; *p* = 0.4) (Figure A4b).

#### 3.4.6. Robotic-Assisted Versus Navigation-Assisted

Including heterogeneous studies (I^2^ = 74.5%), the pooled analysis revealed no significant difference between the robotic-assisted and navigation-assisted groups (SMD: 0.19 with 95% CI [−4.64, 4.83], *p* = 0.93) (Figure 5b).

#### 3.4.7. Meta-Regression

**Figure 7:**
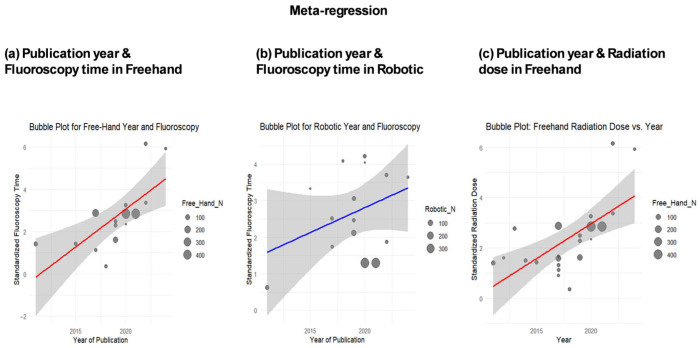
Results of meta-analysis. Meta-regression of fluoroscopy time in the freehand group (**a**), fluoroscopy time in the robotic group (**b**), and radiation dose in the freehand group (**c**).

**Figure A5:**
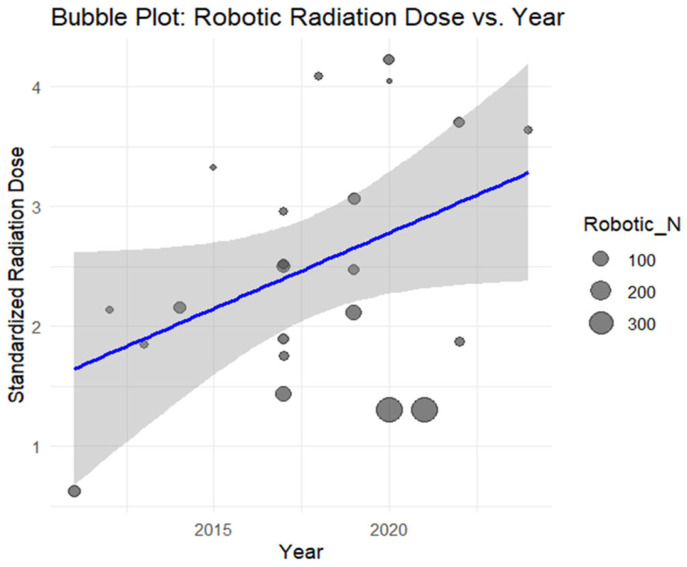
Meta-regression: Publication year and radiation dose in the robotic-assisted group.

**Figure A6:**
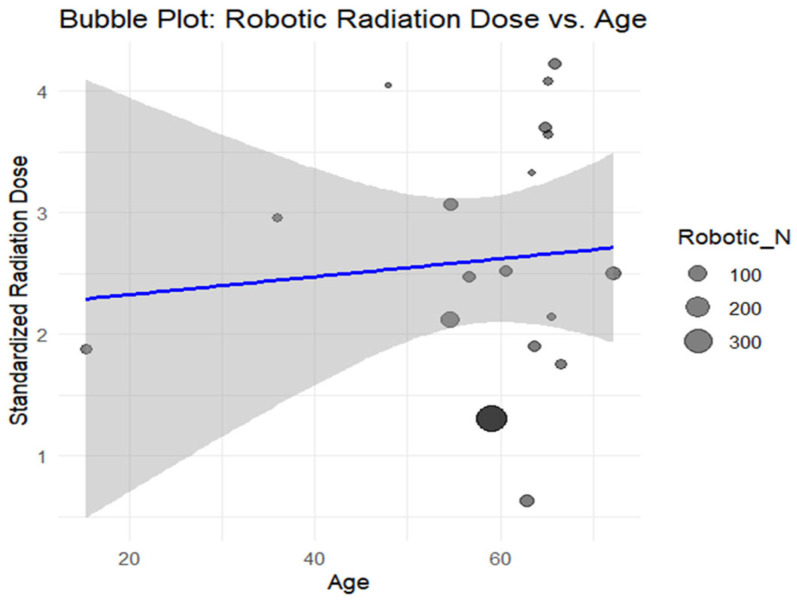
Meta-regression: Age and radiation dose in robotic-assisted group.

**Figure A7:**
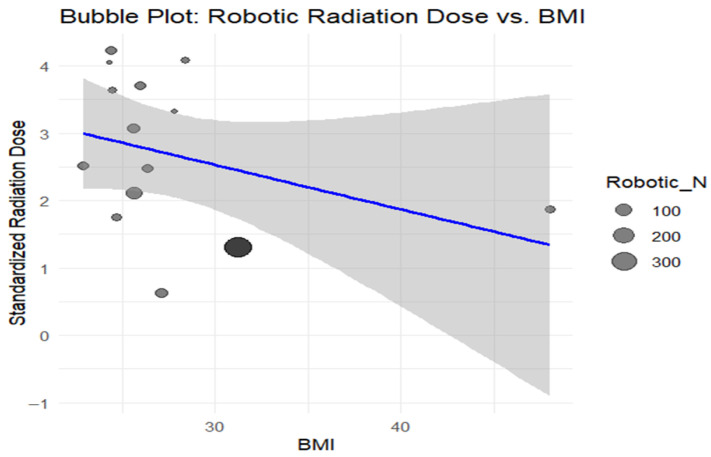
Meta-regression: BMI and radiation dose in the robotic group.

**Figure A8:**
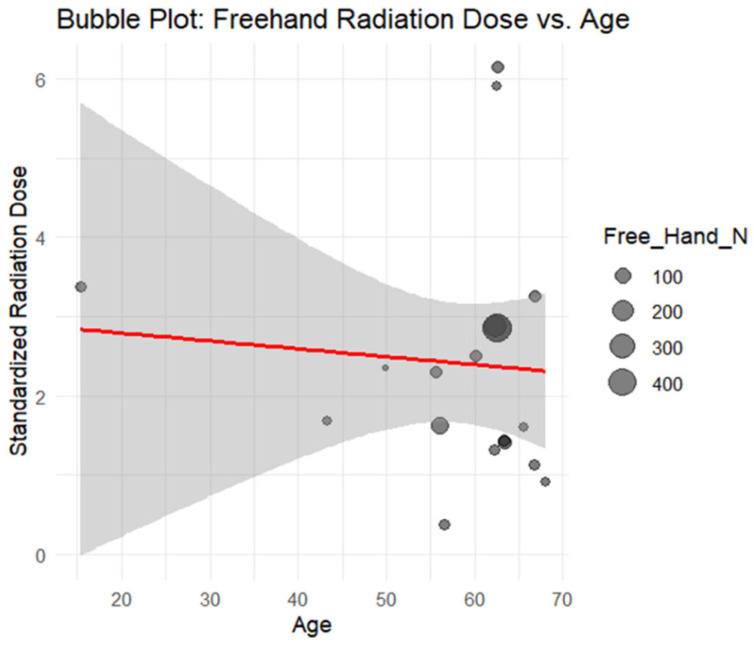
Meta-regression: Age and radiation dose in the freehand group.

**Figure A9:**
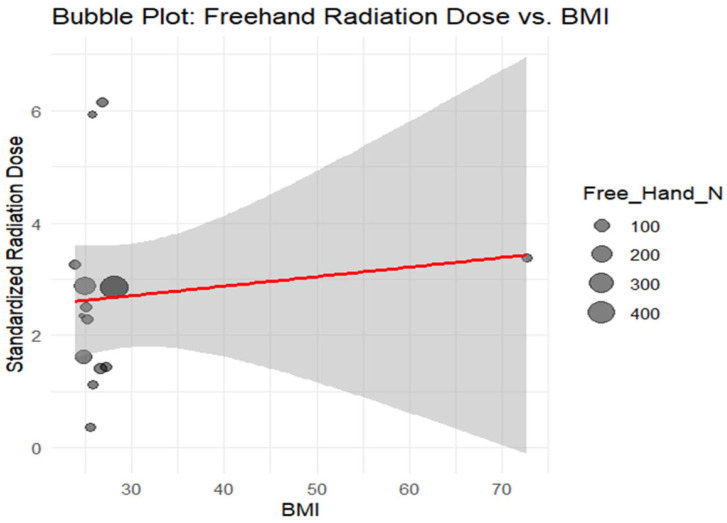
Meta-regression: BMI and radiation dose in the freehand group.

**Figure A10:**
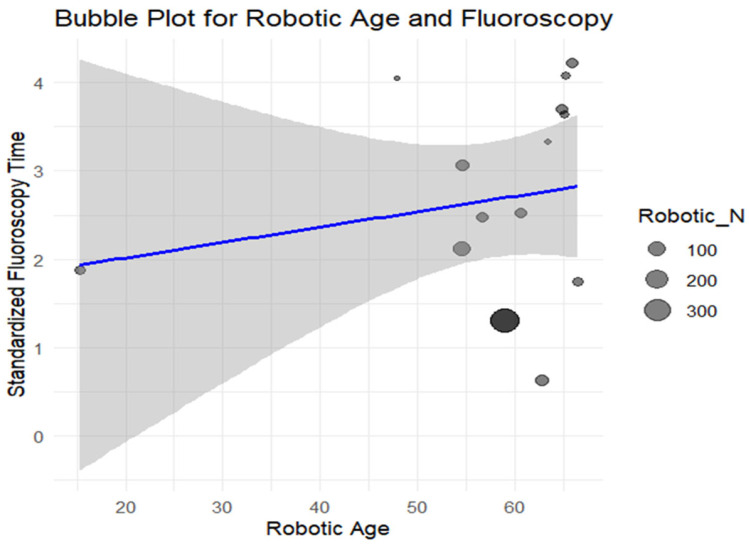
Meta-regression: Age and fluoroscopy time in the robotic-assisted group.

**Figure A11:**
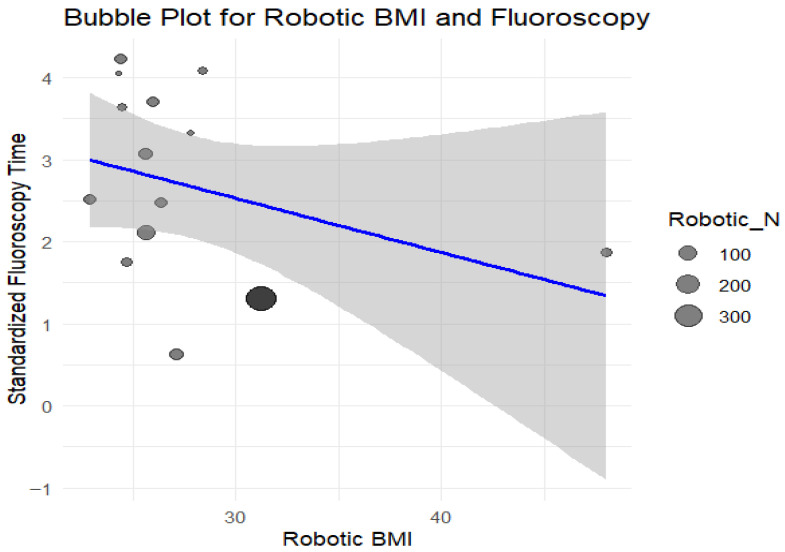
Meta-regression: BMI and fluoroscopy time in the robotic-assisted group.

**Figure A12:**
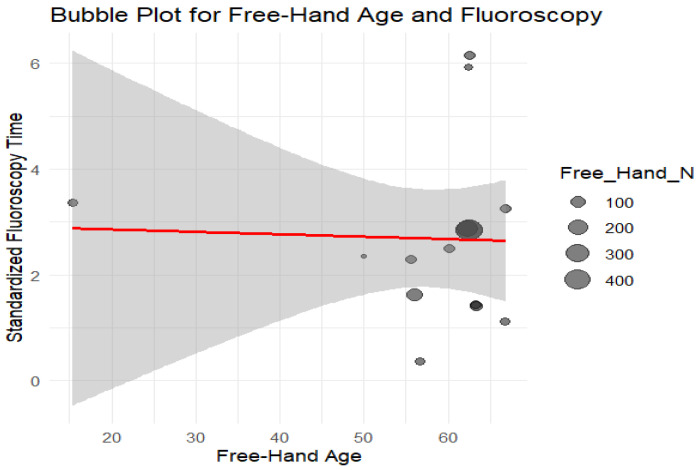
Meta-regression: Age and fluoroscopy time in the freehand group.

**Figure A13:**
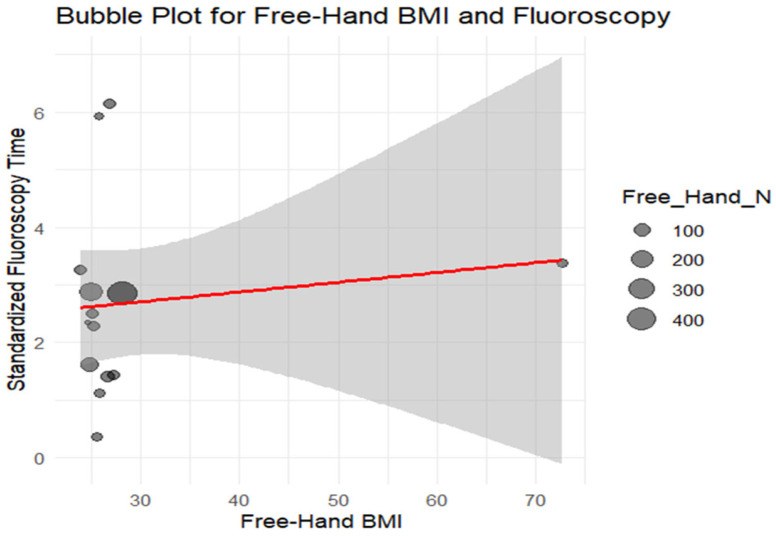
Meta-regression: BMI and fluoroscopy time in the freehand group.

Publication year significantly increased fluoroscopy time in the freehand group (β = 0.3897, *p* = 0.0001) (Figure 7a) and showed a non-significant upward trend in the robotic group (β = 0.1715, *p* = 0.06) (Figure 7b). Radiation dose increased with freehand (β = 0.3930, *p* = 0.014) but not robotics techniques. Age and BMI were non-significant (Figure A5, Figure A6, Figure A7, Figure A8, Figure A9, Figure A10, Figure A11, Figure A12 and Figure A13).

#### 3.4.8. Publication Bias

**Figure A14:**
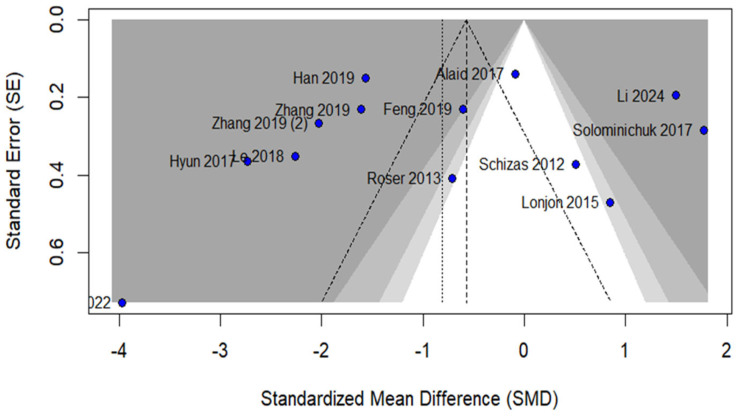
Funnel plot for radiation dose.

**Figure A15:**
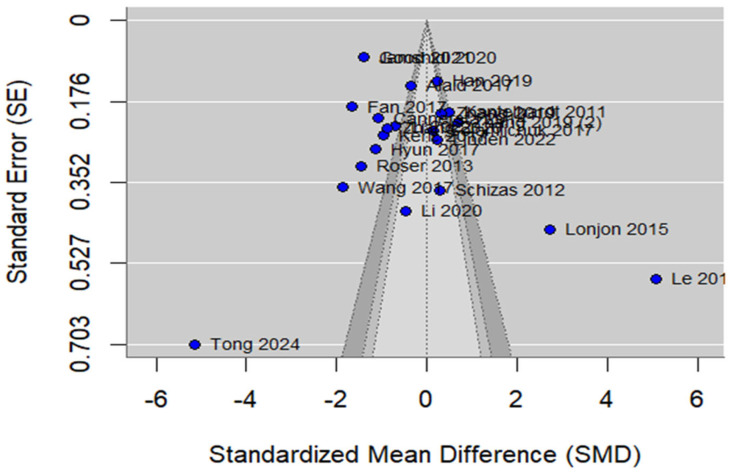
Funnel plot for fluoroscopy time.

By inspecting the funnel plots and Egger’s test results, there was no significant publication bias regarding the radiation dose (Z = −1.3444, *p* = 0.1788) (Figure A14) or fluoroscopy time (Z= 0.3019, *p* = 0.7627) (Figure A15).

## 4. Discussion

Radiation exposure during spinal surgery poses risks to patients and OR staff, with recommended limits of 5 rem for total-body radiation and 50 rem for extremities per year [47]. Exposure varies with surgeon and technician experience, and safe fluoroscopy practices keep annual doses below recommended limits [48]. Transitioning from freehand to robot-assisted pedicle screw placement enhances precision, reduces complications, and improves outcomes [23,25,26,35,36,37]. Early studies, including that of Kantelhardt and Devito (2011) [40], demonstrated feasibility and accuracy, highlighting robotic surgery’s potential to lower radiation exposure and prevent screw misplacement [49].

### 4.1. Summary of Results and Justifications

The pooled analysis revealed no significant difference in radiation dose between the robotic-assisted (RA) and freehand (FH) groups (*p* = 0.07; I^2^ = 96.9%), though the high heterogeneity likely arose from differences in anatomical level, surgical setting, and robot type. The effect direction (SMD −0.81) suggested a potential RA advantage. Leave-one-out sensitivity analyses excluding Solomiichuk et al. [43] or Li et al. [25] demonstrated a significant RA reduction, indicating that specific studies may mask robotic benefits.

Subgroup analyses identified robot type, clinical indication, anatomical level, and region as key determinants. The Tianji^®^ system (Tinavi) showed significantly lower radiation exposure than FH (SMD −1.44; 95% CI −2.64–−0.25), consistent with advanced Chinese robotic systems optimizing workflow and minimizing intraoperative imaging [50]. RA techniques reduced radiation in degenerative disease, while FH was favored in deformity/degenerative and neoplastic cases, likely due to increased fluoroscopic confirmation in complex anatomy. Robotic benefits are greatest in typical degenerative patients with standard anatomy [11]. Zhang et al. (2019) [34] reported FH radiation to be 2.16 times higher than that of RA techniques during lumbar pedicle screw placement (65.3 ± 28.3 vs. 30.3 ± 11.3 μSv).

Anatomical stratification showed robotic assistance significantly reduced radiation exposure in the cervical, lumbar, lumbosacral, and thoracolumbar regions, especially in deeper or junctional zones. Conversely, a FH technique was favored in thoracic regions, possibly due to study limitations grouping the thoracic and lumbar levels, surgical inexperience, or less-frequent procedures in these areas. These findings highlight that RA benefits vary with anatomy, surgeon experience, and case selection. Robotic precision improves outcomes in complex regions, but workflow delays may offset radiation dose advantages [42].

Regional analysis showed lower radiation in Asian studies, particularly China, where high surgical volume and rapid learning curves may reduce exposure [28]. RA and navigation-assisted techniques showed no significant dose difference. Villard et al. (2014) [51] found that surgeon exposure was nearly tenfold higher with 2D fluoroscopy versus navigation. Bratschitsch et al. (2019) [52] demonstrated navigation significantly reduced team radiation exposure (49 ± 19 vs. 566 ± 560 μSv), allowing surgeons to perform up to tenfold more procedures before reaching annual limits.

Overall, RA reduces radiation exposure variably depending on robot type, anatomical complexity, clinical indication, surgeon experience, and regional practice patterns, while navigation also offers substantial protection compared with freehand techniques.

The pooled analysis indicated a marginal trend favoring robotic-assisted (RA) surgery in fluoroscopy time (SMD −0.56; 95% CI −1.15–0.03; *p* = 0.06), which became significant after excluding Le et al., Lonjon et al., or Zhang et al., suggesting RA may improve intraoperative efficiency despite high heterogeneity [21]. Jamshidi et al. (2021) [21] reported a 78.3% reduction in total fluoroscopy time and a 79.8% reduction per screw with RA, corresponding to a 50.8% reduction in overall operative fluoroscopy.

The subgroup analyses highlighted differences by robot type. Renaissance^®^ and SpineAssist^®^ significantly reduced fluoroscopy time due to advanced preoperative planning and percutaneous workflows [53,54], likely reflecting their longer market presence and accumulated surgical experience. Conversely, ROSA^®^ favored freehand techniques, potentially due to workflow complexity or operator learning curves. Clinical indications such as trauma and pyogenic disease showed substantial RA benefits, as robotic precision expedites urgent fixation and reduces repositioning errors. Anatomically, RA techniques decreased fluoroscopy in sacroiliac regions but increased it at the lumbosacral junction, likely reflecting registration challenges in transitional zones. Despite more extensive instrumentation, RA techniques required fewer fluoroscopic spot checks than freehand procedures.

Good et al. (2021) [22] reported per-screw fluoroscopy times of 3.6 ± 3.9 s with Mazor versus 17.8 ± 9.0 s in freehand procedures, an 80% reduction (*p* < 0.001), and the total intraoperative exposure was less than half that of the freehand group (74.8 ± 57.3 vs. 151.9 ± 53.1 s, *p* < 0.001). Wang et al. (2017) [26] similarly reported reduced radiation with RA sacroiliac screw fixation. Regionally, USA studies showed significant RA reductions, likely due to advanced systems and institutional experience, though subgroup differences were not significant (*p* = 0.4).

Meta-regression indicated increasing fluoroscopy time and radiation exposure over time for freehand procedures, reflecting more complex cases, increased 3D imaging use, or higher accuracy demands. RA techniques showed a non-significant rising trend, suggesting workflow improvements mitigate exposure increases. Comparisons between RA and navigation-assisted groups showed no significant differences, consistent with Roser et al. (2013) [10] and Fan et al. (2018) [27].

Overall, robotic assistance provides significant benefits in degenerative cases and specific anatomical regions, such as the cervical and lumbosacral levels. Renaissance^®^ demonstrates the greatest reduction in fluoroscopy time, corroborating Kantelhardt et al. [10,40] and Roser et al. [10]. Meta-regression revealed radiation exposure has increased over time in freehand procedures but remained more consistent with RA procedures, highlighting the impact of robotic precision, technical maturation, and experience [11,50,51]. Across most subgroup strata, the heterogeneity remained substantial, indicating that subgrouping did not fully explain between-study variability; therefore, these subgroup findings should be interpreted as exploratory and hypothesis-generating rather than definitive.

### 4.2. Limitations

Despite its rigorous methodology, this study has notable limitations. First, outcome measurement was not uniform across studies: radiation was reported using different dose metrics/units, and fluoroscopy time was reported using different bases (e.g., per screw vs. per procedure), which likely contributed to substantial heterogeneity and may introduce detection bias. Second, imaging workflows and operative protocols (e.g., use of 2D fluoroscopy vs. 3D imaging, institutional radiation practices) varied across settings and were not consistently reported. Third, many of the included studies were retrospective, increasing susceptibility to selection bias and residual confounding (e.g., surgeon experience and learning-curve effects, case complexity, and center-specific protocols). Finally, the persistence of heterogeneity reduces certainty and limits generalizability, supporting the need for prospective studies with standardized radiation and fluoroscopy reporting.

## 5. Conclusions

In pooled analyses, robotic-assisted surgery was not associated with a statistically significant reduction in overall radiation dose or fluoroscopy time compared with freehand techniques. Exploratory subgroup and sensitivity analyses suggested possible differences across robotic platforms and clinical contexts; however, substantial heterogeneity and variable outcome reporting limit generalizability. Standardized prospective comparative studies are needed.

## Figures and Tables

**Table 1:** Summary of included studies.

Study ID	Study Design	N. of Centers (Single-Center vs. Multi-Center)	Country	Sample Size	Clinical Indication/Anatomical Level	Robot Type	Main Inclusion Criteria	Key Radiation Exposure Findings
Alaid et al., 2018 [39]	Retrospective cohort	Single-center	Germany	206	IN/L	SpineAssist^®^ (Mazor Robotics)	Patients with pyogenic spondylodiscitis need instrumentation.	The RG group had a lower mean fluoroscopy time (123 ± 86 s vs. 157 ± 99 s, *p* = 0.014) and a trend toward a lower radiation dose.
Feng et al., 2019 [24]	Randomized controlled trial	Single-center	China	80	DG/L	Tianji^®^ (Tinavi)	Age ≥ 50, bone density < 80 mg/cm^3^, degenerative lumbar disease, pedicle screw fixation.	The RG had significantly lower radiation exposure (38.87 ± 11.94 mSv vs. 47.45 ± 15.75 mSv, *p* < 0.05).
Fan et al., 2017 [23]	Retrospective cohort	Single-center	China	190	DG/L	SpineAssist^®^ (Mazor Robotics)	Adults undergoing 3-column reconstruction for degenerative spine disease.	The RG had the shortest fluoroscopy time per screw (4.02 ± 1.6 s) compared to the NT (1.29 ± 0.6 s), O-arm navigation (6.36 ± 1.7 s), and fluoroscopy-guided (8.89 ± 3.1 s) groups.
Good et al., 2021 [22]	Randomized controlled trial	Multi-center	USA	485	DG/L, LS	Renaissance^®^ (Mazor Robotics)	Adults ≥ 21, scheduled for primary short lumbar/lumbosacral fusion.	Fluoroscopy time per screw: 15.5 s RG vs. 35.4 s FG. During instrumentation: 3.6 s RG vs. 17.8 s FG (80% reduction, *p* < 0.001).
Han et al., 2019 [28]	Randomized controlled trial	Single-center	China	234	DG, TF/TL	Tianji^®^ (Tinavi)	Age 18–80, thoracolumbar fusion.	Radiation time: 81.5 ± 38.6 s RG vs. 71.5 ± 44.2 s FG (*p* = 0.07). Surgeon exposure: 21.7 ± 11.5 μSv RG vs. 70.5 ± 42.0 μSv FG (*p* < 0.01).
Hyun et al., 2017 [44]	Randomized controlled trial	Single-center	Korea	60	DG/L	Renaissance^®^ (Mazor Robotics)	Single-/double-level lumbar disorders for interbody fusion.	Fluoroscopy use was 3.5 s in RG vs. 13.3 s in FG (*p* < 0.001). C-arm output was 0.13 mSv in RG vs. 0.27 mSv in FG (*p* = 0.015). Per-screw radiation in RG was 37.5% of FG, showing a 62.5% reduction.
Kantelhardt et al., 2011 [40]	Retrospective cohort	Single-center	Germany	112	DG/L, T, S	SpineAssist^®^ (Mazor Robotics)	Pedicle screw placement.	Intraoperative X-ray exposure was 34 s in RG procedures compared to 77 s in conventional cases.
Keric et al., 2017 [41]	Retrospective cohort	Single-center	Germany	90	IN/L, T	Renaissance^®^ (Mazor Robotics)	Pyogenic lumbar/thoracic spondylodiscitis.	The average X-ray exposure per screw was 0.94 ± 1.04 min in the FG group versus 0.4 ± 0.16 min in the RG group (*p* = 0.000).
Le et al., 2018 [29]	Retrospective matched cohort	Single-center	China	58	DG/L	Tianji^®^ (Tinavi)	CBT screw instrumentation.	The RG group had a longer radiation time (142.8 s vs. 77.5 s) but significantly lower radiation exposure to the surgeon.
Li et al., 2020 [30]	Randomized controlled trial	Single-center	China	17	DG/L	ORTHBOT^®^ (Shenzhen Futurtec)	Age 18–65, posterior fusion for lumbar disk disease/spinal stenosis.	In comparing CBT screw placement, the RG group had a shorter radiation time (0.37 min vs. 0.43 min), a statistically significant difference.
Li et al., 2022 [31]	Retrospective cohort	Single-center	China	81	DG/L	Tianji^®^ (Tinavi)	Age ≤ 85, degenerative lumbar disease needing cortical screw technique.	The RG group had a shorter radiation time (0.37 ± 0.10 min) than the FG group (0.43 ± 0.07 min, *p* = 0.001).
Lonjon et al., 2016 [46]	Prospective cohort	Single-center	France	20	DG/L	ROSA Spine^®^ (Medtech)	Age 18–80, posterior fusion for degenerative lumbar disease.	The RG group had a longer fluoroscopy time (1.23 min vs. 0.40 min) and higher radiation exposure (821 cGy cm^2^ vs. 406 cGy cm^2^) compared to the FG group.
Roser et al., 2013 [10]	Prospective randomized study	Single-center	Germany	148	TF/T, L	SpineAssist, Mazor Robotics	Participants were over 18, needed single-level lumbar stabilization with pedicle screws, and had no prior spine surgery at the affected level.	Navigation-assisted surgery had 10.36 s and 4.04 mGy of radiation per screw, RG had 15.98 s and 11.03 mGy of radiation per screw, while FG had 31.5 s and 18.9 mGy of radiation per screw.
Ringel et al., 2012 [42]	Randomized controlled trial	Single-center	Germany	60	DG/LS	SpineAssist^®^ (Mazor Robotics)	Age > 18, lumbosacral stabilization using pedicle screws.	1.9 min intraoperative radiation time for both groups, plus an additional 411.6 mGy cm CT radiation for robot-assisted cases only.
Schizas et al., 2012 [45]	Prospective cohort	Single-center	Switzerland	34	DG, TF/T, L, S	Renaissance^®^ (Mazor Robotics)	Adults requiring pedicle screw instrumentation.	16.7 vs. 14.2 s duration and 0.18 vs. 0.11 mGy/m^2^ dose for RG vs. FG groups.
Solomiichuk et al., 2017 [43]	Retrospective cohort	Single-center	Germany	70	NP/T, TL	SpineAssist^®^ (Mazor Robotics)	Metastatic spinal disease requiring instrumentation.	138.2 vs. 126.5 s duration and 2.8 vs. 2.0 mAs intensity for RG vs. FG groups.
Tong et al., 2024 [32]	Retrospective cohort	Single-center	China	38	DG/L	Mazor X^®^ (Mazor Robotics)	Single-segment lumbar degenerative disease (LDD) needing OLIF with PPS fixation.	13.31 vs. 48.45 fluoroscopy times for RG vs. FG groups.
Zhan et al., 2022 [33]	Retrospective cohort	Single-center	China	25	DD, TF/C	Tianji^®^ (Tinavi)	Atlantoaxial dislocation (AAD) needing posterior C1–C2 screw fixation.	Radiation exposure: 12.4 vs. 19.9 mGy/screw for RG vs. FG groups.
Zhang et al., 2019 [34]	Prospective cohort	Multi-center	China	100	DG/L	Tianji^®^ (Tinavi)	Lumbar degenerative disease (L1–S1) with radiculopathy, failed conservative treatment.	Radiation exposure: 30.3 vs. 65.3 μSv for RG vs. FG groups.
Zhang et al., 2019 (2) [20]	Prospective cohort	Single-center	China	77	DG/L	Tianji^®^ (Tinavi)	Lumbar degenerative disease needing TLIF.	The radiation dose was 25.9 vs. 70.5 mSv for RG vs. FG groups.
Zhang et al., 2020 [35]	Retrospective cohort	Single-center	China	81	DG, TF/TL, L	Renaissance^®^ (Mazor Robotics)	Revision lumbar surgery with pedicle screws implanted by robot or freehand.	2.32 vs. 2.99 s fluoroscopy time for RG vs. FG groups.
linden et al., 2022 [36]	Retrospective cohort	Single-center	USA	60	DF/T, TL, L	Mazor X^®^ (Mazor Robotics)	Adolescent idiopathic scoliosis (AIS), posterior fusion surgery.	The RG arm reduced fluoroscopy time per screw to 4.02 s, compared to 1.29 s for navigation template, 6.36 s for O-arm navigation, and 8.89 s for fluoroscopy-guided methods.
Jamshidi et al., 2021 [21]	Retrospective cohort	Multi-center	USA	485	DG/L	Renaissance^®^ (Mazor Robotics^®^)	Patients requiring short-segment (1–3 level) minimally invasive lumbar fusion.	RG pedicle screw placement reduced fluoroscopy time by 78.3% compared to FG placement.
Khan et al., 2020 [37]	Retrospective cohort	Single-center	USA	40	DG/L	Mazor X^®^ (Mazor Robotics)	Degenerative disk disease with spondylolisthesis needing stabilization.	RG fluoroscopy time: 10.1 ± 5.4 s, radiation dose: 66.2 ± 38.1 mGy, CT navigation fluoroscopy time: 11.2 ± 9.4 s, radiation dose: 42.9 ± 34.8 mGy
Li et al., 2024 [25]	Retrospective cohort	Single-center	China	144	DG, DF/L, C	Tianji^®^ (Tinavi)	Scoliosis correction, Cobb angle < 90°, flexibility indices > 30%.	Radiation exposure: RG O-arm: 39.18 μSv, 3D C-arm: 4.85 μSv, FG technique: 15.97 × 10^−5^ μSv.
Wang et al., 2017 [26]	Randomized controlled trial	Single-center	China	30	TF/SI	Tianji^®^ (Tinavi)		The RG group had significantly shorter fluoroscopy times (6 s vs. 36 s) compared to the FG group (*p* < 0.001).
Fan et al., 2018 [27]	Retrospective cohort	Single-center	China	267	DG/L	Renaissance^®^ (Mazor Robotics^®^)	Severe adult degenerative scoliosis, with greater rotational deformity and lordosis loss.	The RG arm reduced fluoroscopy time per screw to 4.02 s, compared to 1.29 s for navigation template, 6.36 s for O-arm navigation, and 8.89 s for FG methods.
Cannestra et al., 2014 [38]	Retrospective cohort	Single-center	USA	102	DG/NA	N/A	N/A	The RG arm significantly reduced fluoroscopic radiation exposure per screw (27.66 s) compared to FG (60.76 s) and the freehand percutaneous technique (64.16 s).

Abbreviations: C, Cervical; DF, deformity; DG, degenerative diseases; FG, freehand guidance conventional technique; IN, infectious disease; L, lumbar; LS, lumbosacral; N/A, not available; NP, neoplastic disease; RG, robotic-guided; S, sacral; SI, sacroiliac; T, thoracic; TF, traumatic/fracture; TL, thoracolumbar.

**Table 2:** Baseline characteristics.

Study ID	Study Arm	Number of Patients	Number of Screws	Age (Years), Mean ± SD	Sex (Female), N (%)	BMI (kg/m^2^), Mean ± SD	Type of Intervention or Surgery
Alaid et al., 2018 [39]	Robotic	98	N/A	N/A	N/A	N/A	Minimally invasive percutaneous pedicle screw placement
	Freehand	108	N/A	N/A	N/A	N/A	Open pedicle screw placement (fluoroscopy-guided)
Fan et al., 2017 [23]	Robotic	39	176	60.6 ± 7.9	20 (51%)	22.9 ± 4.7	Minimally invasive percutaneous pedicle screw placement (decompression or TLIF)
	Freehand	72	346	62.4 ± 8.9	39 (54%)	25.0 ± 7.3	Pedicle screw placement (decompression or TLIF) Open pedicle screw placement (fluoroscopy-guided) (decompression or TLIF)
	O-arm-based navigation	51	234	65.1 ± 8.0	31 (61)	24.8 ± 3.5	O-arm navigation system
Feng et al., 2019 [24]	Robotic	40	202	67.55 ± 6.50	28 (70%)	24.94 ± 4.52	Robot-assisted pedicle screw insertion
	Freehand	40	225	67.88 ± 7.34	27 (67.5%)	25.55 ± 3.46	Fluoroscopy-assisted freehand pedicle screw insertion
Good et al., 2021 [22]	Robotic	374	1813	59.0 ± 12.6	208 (56.1%)	31.2 ± 6.8	Minimally invasive percutaneous/MIS spinal fusion with Mazor Core technology
	Freehand	111	484	62.5 ± 12.8	69 (62.2%)	28.1 ± 5.2	Minimally invasive spinal fusion with fluoroscopic guidance
Han et al., 2019 [28]	Robotic	115	532	54.6 ± 11.3	60(52.2%)	25.7 ± 4.1	Pedicle screw insertion (thoracolumbar)
	Freehand	119	584	56.1 ± 13.4	61 (51.3%)	24.9 ± 2.9	Pedicle screw insertion (thoracolumbar)
Hyun et al., 2017 [44]	Robotic	30	130	66.5 ± 8.1	21 (70%)	24.7 ± 2.6	Minimally invasive robotic-guided spinal instrumented fusion
	Freehand	30	140	66.8 ± 8.9	22 (73.3%)	25.8 ± 3.3	Open fluoroscopic-guided spinal instrumented fusion
Kantelhardt et al., 2011 [40]	Robotic	55	250	62.8 ±11.23	30 (55%)	31.3 ± 8.1	Open or percutaneous pedicle screw placement with robotic guidance
	Freehand	57	286	63.4 ±11	30(52.6%)	27 ± 3.7	Open pedicle screw placement with 2D fluoroscopic guidance
Keric et al., 2017 [41]	Robotic	24	341	72.3 11.1	30 (45.5%)	N/A	Percutaneous pedicle screw placement with robotic guidance
	Freehand	66	121	68 ± 11.23	11(45.8%)	N/A	Open pedicle screw placement with fluoroscopic guidance
Le et al., 2018 [29]	Robotic	20	86	65.2 ± 8.5	14 (70%)	28.4 ± 3.6	Cortical bone trajectory (CBT) screw instrumentation
	Freehand	38	145	56.6 ± 14.8	26 (68.4%)	25.5 ± 3.8	
Li et al., 2020 [30]	Robotic	7	32	47.4 ± 12.9	4 (57.1%)	24.3 ± 1.8	Posterior lumbar interbody fusion with pedicle screw fixation
	Freehand	10	50	49.9 ± 10.9	6 (60%)	24.6 ± 2.6	
Li et al., 2022 [31]	Robotic	37	172	64.86 ± 9.70	23 (62.2%)	25.99 ± 4.20	Cortical bone trajectory (CBT) screw placement for degenerative lumbar spine disease
	Freehand	44	204	62.64 ± 11.13	26 (59.1%)	26.90 ± 4.53	
Lonjon et al., 2016 [46]	Robotic	10	40	63.4 ± 11	6 (60%)	27.8 ± 4	Open posterior median approach with pedicle screw implantation, ±laminectomy, and/or inter-somatic fusion (cage)
	Freehand	10	50	63.4 ± 11	6 (60%)	27.3 ± 5.6	
Ringel et al., 2012 [42]	Robotic	60	146	68	16 (55%)	26	Open approach with pedicle screw implantation, ±decompression, or fusion
	Freehand		152	67	18 (62%)	28	
Roser et al., 2013 [10]	Robotic	18	72	N/A	N/A	N/A	Open or percutaneous pedicle screw implantation, ±decompression or fusion
	Freehand	10	40	N/A	N/A	N/A	N/A
	Navigation	9	36	N/A	N/A	N/A	Open surface-matching spinal neuro-navigation
Schizas et al., 2012 [45]	Robotic	11	64	65.5	5 (45.5%)	N/A	Open pedicle screw insertion
	Freehand	23	64	65.5	22 (95.7%)	N/A	
Solomiichuk et al., 2017 [43]	Robotic	35	192	63.7	12 (34.3%)	N/A	Posterior instrumentation (open vs. minimally invasive)
	Freehand	35	214	62.2	12 (34.3%)	N/A	
Tong et al., 2024 [32]	Robotic	16	64	65.1	7 (43.8%)	24.5	(Fluoroscopic) OLIF with PPS fixation
	Freehand	22	88	62.6	13 (59.1%)	25.8	
Zhan et al., 2022 [33]	Robotic	11	44	50.6	5 (45.5%)	23.3	Posterior C1–C2 screw fixation (Goel–Harms technique)
	Freehand	14	56	55.4	4 (28.6%)	21.3	
Zhang et al., 2019 [34]	Robotic	50	100	54.6 ± 11.1	33 (66%)	25.6 ± 3.9	Transforaminal lumbar interbody fusion (TLIF)
	Freehand	50	100	55.6 ± 12.8	29 (58%)	25.3 ± 3.1	
Zhang et al., 2019 (2) [20]	Robotic	43	176	56.7	31 (72.1%)	26.4	Transforaminal lumbar interbody fusion (TLIF)
	Freehand	44	204	60.2	26 (59.1%)	25.2	
Zhang et al., 2020 [35]	Robotic	39	267	65.95 ± 7.55	18 (46%)	24.46 ± 2.26	Posterior lumbar revision surgery with pedicle screw implantation
	Freehand	42	288	66.86 ± 7.32	20 (48%)	23.68 ± 2.58	
linden et al., 2022 [36]	Robotic	30	17	15 ±2.01	23 (77%)	48 ± 30	Posterior spinal fusion with pedicle screw placement using robotic-assisted navigation
	Freehand	30	13	15.3 ±1.9	23 (77%)	72.6 ± 31.1	Posterior spinal fusion with pedicle screw placement using the freehand technique.
Jamshidi et al., 2021 [21]	Robotic	374	1795	59.0± 12.6	210 (56.1%)	31.2 ± 6.8	Minimally invasive short-segment (1–3 levels) lumbar fusion using robotic guidance (Mazor Renaissance^®^ system) for pedicle screw placement
	Freehand	111	488	62.5 ± 12.8	69 (62.2%)	28.1 ± 5.2	Minimally invasive short-segment (1–3 levels) lumbar fusion using standard 2D fluoroscopy for pedicle screw placement
Khan et al., 2020 [37]	Robotic	22	92	65.2± 9.9	16(72.7%)	28.4 ± 4.2	CBT pedicle screw insertion with robotic guidance
	CT-based navigation	18	74	63.7± 7.3	14(77.8%)	29.1± 4.6	CBT pedicle screw insertion with 3D CT navigation
Li et al., 2024 [25]	Robotic	92	1080	32.2 ± 11	68 (74%)	21.9 ± 2.8	Scoliosis correction with pedicle screws
	Freehand	52	722	29.1 ± 12.139	35 (67%)	22.4 ± 3.1	
Wang et al., 2017 [26]	Robotic	23	23	36	5 (21.7%)	N/A	Robot-assisted percutaneous SI screw fixation using TiRobot
	Freehand	22	22	43	7 (31.8%)	N/A	Conventional fluoroscopy-assisted freehand percutaneous SI screw fixation
Fan 2018 [27]	Robotic	83	1012	61.6 ± 9.1	48 (58%)	25.8 ± 3.6	Open robot-assisted posterior lumbar interbody fusion (PLIF) surgery
	CT-based navigation	109	1276	63.9 ± 8.4	65 (60%)	27.3 ± 3.9	CT-based navigation system-assisted PLIF surgery
Cannestra 2014 [38]	Robotic	51	280	NA	NA	NA	Percutaneous robotic guidance
	Freehand	51	270	NA	NA	NA	Freehand technique, in either a percutaneous (N540) or open surgical approach (N511)

Abbreviations: BMI, Body mass index; CT, computed tomography; NA, not available; SD, standard deviation.

## Data Availability

The data presented in this study are available on request from the corresponding authors.

## Supplementary Materials

The following supporting information can be downloaded at https://www.mdpi.com/article/10.3390/jcm15062144/s1, Table S1: PRISMA checklist.

## Abbreviations

The following abbreviations are used in this manuscript:

BMI	Body Mass Index
CT	Computed Tomography
MIS	Minimally Invasive Surgery
NA	Navigation-Assisted
FH	Freehand
RA	Robotic-Assisted
RCT	Randomized Controlled Trial
TLIF	Transforaminal Lumbar Interbody Fusion

## Appendix A

**Table A1:** Search terms and search results.

Database	Search Term	Search Field	Results
PubMed	(robot* OR “robotic surgery”) AND (spine OR spinal) AND (radiation OR fluoroscopy) AND (surgery OR procedure)	ALL Fields	576
WOS	(robot* OR “robotic surgery”) AND (spine OR spinal) AND (radiation OR fluoroscopy) AND (surgery OR procedure)	ALL Fields	568
Scopus	(robot* OR “robotic surgery”) AND (spine OR spinal) AND (radiation OR fluoroscopy) AND (surgery OR procedure)	ALL Fields	835
Cochrane	(robot* OR “robotic surgery”) AND (spine OR spinal) AND (radiation OR fluoroscopy) AND (surgery OR procedure)	All Text	49

**Table A2:** Assessment of the quality of studies through the Methodological Index for Non-Randomized Studies (MINORS).

Study ID	Clearly Stated Aim	Consecutive Patients	Prospective Collection of Data	Endpoints	Assessment of Endpoint	Follow-Up Period	Loss < 5%	Study Size	Adequate Control Group	Contemporary Group	Baseline Control	Statistical Analyses	MINORS Score
Alaid et al., 2018 [39]	2	2	1	2	2	2	2	2	2	2	2	2	18
Cannestra, 2014 [38]	2	2	1	2	2	2	2	2	2	2	2	2	19
Fan, 2018 [27]	2	2	1	2	2	2	2	2	2	2	2	2	18
Fan et al., 2017 [23]	2	2	1	2	2	2	2	2	2	2	2	2	20
Jamshidi et al., 2021 [21]	2	2	1	2	2	2	2	2	2	2	2	2	17
Kantelhardt et al., 2011 [40]	2	2	1	2	2	2	2	2	2	2	2	2	19
Keric et al., 2017 [41]	2	2	1	2	2	2	2	2	2	2	2	2	18
Khan et al., 2020 [37]	2	2	1	2	2	2	2	2	2	2	2	2	20
Le et al., 2018 [29]	2	2	1	2	2	2	2	2	2	2	2	2	17
Li et al., 2022 [31]	2	2	1	2	2	2	2	2	2	2	2	2	19
Li et al., 2024 [25]	2	2	1	2	2	2	2	2	2	2	2	2	20
Linden et al., 2022 [36]	2	2	1	2	2	2	2	2	2	2	2	2	18
Lonjon et al., 2016 [46]	2	2	1	2	2	2	2	2	2	2	2	2	19
Schizas et al., 2012 [45]	2	2	1	2	2	2	2	2	2	2	2	2	17
Solomiichuk et al., 2017 [43]	2	2	1	2	2	2	2	2	2	2	2	2	20
Tong et al., 2024 [32]	2	2	1	2	2	2	2	2	2	2	2	2	18
Zhan et al., 2022 [33]	2	2	1	2	2	2	2	2	2	2	2	2	19
Zhang et al., 2019 [34]	2	2	1	2	2	2	2	2	2	2	2	2	20
Zhang et al., 2019 (2) [20]	2	2	1	2	2	2	2	2	2	2	2	2	18
Zhang et al., 2020 [35]	2	2	1	2	2	2	2	2	2	2	2	2	17

**Table A3:** Study radiation dose measure/unit and fluoroscopy time basis.

Study ID	Radiation Dose Unit	Time Basis
Alaid 2018 [39]	mAs	per case
Cannestra 2014 [38]	NA	per screw
Fan 2017 [23]	NA	per screw
Fan 2018 [27]	mSv	NA
Feng 2019 [24]	μSv	NA
Good 2021 [22]	NA	per case
Han 2019 [28]	μSv	per case
Hyun 2017 [44]	mSv/screw	per screw
Jamshidi 2021 [21]	NA	per case
Kantelhardt 2011 [40]	NA	per case
Keric 2017 [41]	NA	per screw
Khan 2020 [37]	mGy,	per case
Le 2018 [29]	μSv	per case
Li 2020 [30]	NA	per case
Li 2022 [31]	NA	per case
Li 2024 [25]	μSv	NA
Linden 2022 [36]	NA	per case
Lonjon 2016 [46]	cGy·cm^2^	per case
Roser 2013 [10]	mGy	per case
Roser 2013 [10]	mGy	per case
Schizas 2012 [45]	mGy/m^2^	NA
Solomiichuk 2017 [43]	mAs	per case
Tong 2024 [32]	NA	NA
Wang 2017 [26]	NA	per case
Zhan 2022 [33]	mGy/screw	NA
Zhang 2019 [34]	μSv	per case
Zhang 2020 [35]	NA	per screw
Zhang 2019 (2) [20]	μSv	per case
